# Hospital Meetings

**Published:** 1905-02-25

**Authors:** 


### HOSPITAL MEETINGS.

### CHARING CROSS HOSPITAL.

### HANDSOME DONATIONS.

THE eighty-fourth annual meeting of Governors of Charing Cross Hospital took place last week, the chair being taken by Lord Burnham, K.C.V.O.

Referring to the report as an unusually interesting one, since it recorded the successful completion of many remarkable improvements and extensions, the chairman drew attention to the paragraph dealing with the financial position of the Hospital, in which it was stated that the amount required from benevolent sources would be increased this year by £6,225 (of which £1,300 was for increased rates) showing that a sum of at least £23,000 would be required. Under the circumstances, the council had determined to close the north wing of the hospital, when the new wards were opened early in the year, and to postpone the necessary improvements until increased financial support warranted the expenditure. The accounts showed a deficit of £10,714 on the income and expenditure account, which included only amounts received for and expended on the general purposes of the hospital. While the legacies, credited to the capital fund for buildings and equipment, amounted to only £2,663, the council had pleasure in recording the endowment of three beds, two in the Hospital and one in the Convalescent Home. In view of the generally admitted depression of trade and commerce, and the large demands issuing from other kindred institutions for rebuilding, the generous encouragement received, especially from King Edward's Hospital Fund (£2,500, being £1,500 for maintenance and £1,000 for building, the largest grant the hospital had ever received) was most satisfactory.

The report and accounts having been adopted, and the customary votes of thanks passed, Mr. T. P. Borrett, chairman of the hospital, proposed a vote of thanks to Lord Burnham, whose family was closely connected with the support of the institution.

Mr. J. H Morgan, C.V.O., senior surgeon to the hospital, in seconding, said anyone who viewed the outpatients' department would say there was nothing better in the whole of London. The accessories of surgical science were, however, improved upon day by day and year by year, and as an instance of this fact, the theatre, quite the best in London when it was built, was now absolutely out of date. In a few weeks, however, Charing Cross Hospital would have two theatres equal to any in London. He looked upon the financial condition of the hospital very seriously indeed. Fresh improvements meant increased expense; and even if the debts were paid off, there would still be the necessity of raising a larger amount than ever in order to carry on the work of the hospital, while the painful necessity of closing some of the old wards, on the opening of the new ones, had to be faced.

The Chairman, in returning thanks, said that his sisters, Miss Matilda Levy, and Lady Campbell Clarke, desired to place in the hands of the executive the sums of £3,000 and £1,000 respectively, to be devoted to the endowment of four beds in the Levy ward, which would then be completed. The announcement was received with applause.

### CITY OF LONDON LYING-IN HOSPITAL.

The annual Court of Governors was held at the hospital last week. The chairman, Mr. Berry, expressed his pleasure in moving the adoption of the 154th annual report of the hospital. He said that in spite of many drawbacks excellent work had been carried on during the last year. But the time had come when re-building was absolutely necessary. For 154 years the hospital had carried on its good work, but now it was feared that the endowment would be absorbed by the work of re-building, which must be perfectly modern.

Some of those present had perhaps read the newspaper correspondence in which Sir Henry Burdett talked of the "abuse of hospitals." To this the Hon. Sydney Holland objected on the ground that the poor received always the first attention. That was quite true, but still there were people who, in spite of everything, would use special free hospitals, though well able to afford to pay at least part of their maintenance. These could never be shut out, for they would always beg a letter from one governor or another. During the time of building the committee would have to decide if there could not be some paying wards in the hospital, so as to make it in some measure self-supporting. Great things were hoped of the festival dinner to be held on May 23rd, at which the Lord Mayor had consented to preside.

The motion was seconded by Mr. Francis, who, as chairman of the Building Committee, said that they would cut down the estimates and bring them as much as possible to the hospital's means.

The report having been adopted and other business enacted Mr. Gosnell proposed a special motion. It was that the system requiring medical gentlemen to canvass among the Governors might be improved upon. The custom, which was an old-fashioned one, seemed to him derogatory. Some discussion ensued and it was finally decided that any change from the old custom could only be effected by calling a fresh Court. Therefore the chairman said that the motion would have to be brought up in a new form at the next annual meeting. A vote of thanks to the chairman then brought the meeting to a close.

